# Apolipoprotein A5 gene variants and the risk of coronary heart disease: A case-control study and meta-analysis

**DOI:** 10.3892/mmr.2013.1642

**Published:** 2013-08-16

**Authors:** JIANQING ZHOU, LIMIN XU, RONG STEPHANIE HUANG, YI HUANG, YANPING LE, DANJIE JIANG, XI YANG, WEIFENG XU, XIAOYAN HUANG, CHANGZHENG DONG, MENG YE, JIANGFANG LIAN, SHIWEI DUAN

**Affiliations:** 1 Ningbo Medical Center, Lihuili Hospital, Ningbo University, Ningbo, Zhejiang 315041, P.R. China; 2 Zhejiang Provincial Key Laboratory of Pathophysiology, Ningbo University School of Medicine, Ningbo, Zhejiang 315211, P.R. China; 3 Department of Medicine, University of Chicago, Chicago, IL 60637, USA

**Keywords:** coronary heart disease, *APOA5*, SNP, -1131T>C, S19W, 553G>T

## Abstract

Previous studies have shown that apolipoprotein A5 (*APOA5)* gene variants are genetic determinants of the concentration of triglycerides, which are a known risk factor for coronary heart disease (CHD). Using the standardized coronary angiography method, 290 CHD patients and 198 non-CHD controls were recruited from Ningbo Lihuili Hospital. In addition, 331 unrelated healthy volunteers were recruited as healthy controls from Ningbo Ximen Community residents. Three variants of the *APOA5* gene, S19W, -1131T>C and 553G>T, were analyzed for their association with CHD. Under a dominant inheritance model, -1131CT>C was shown to be a CHD risk factor (P=0.030; OR, 1.422; 95% CI, 1.036–1.952). The single nucleotide polymorphism, 553G>T, was found to correlate with the severity of CHD in males (P=0.032). Meta-analysis showed that -1131T>C was significantly associated with CHD (P<0.0001). By contrast, negative correlations with CHD were observed for S19W and 553G>T. In the present case-control study, *APOA5* gene variants were not found to correlate with the risk of CHD in the populations studied; however, -1131CT>C was shown to be a CHD risk factor under a dominant inheritance model. Meta-analysis showed a significant contribution of -1131T>C to the risk of CHD, implying an ethnic difference in *APOA5* gene variants.

## Introduction

Coronary heart disease (CHD) is a severe condition in which plaque builds up inside the coronary arteries. Over time, plaque hardens and narrows the coronary arteries, eventually leading to myocardic infarction and mortality. Triglycerides (TGs) are the major components of plaque and aberrant levels of TGs significantly correlate with the risk of CHD (1). Increased TGs contribute to the development of hypertriglyceridemia and metabolic syndrome (2,3), which are associated with cardiovascular events (2). Although a small number of candidate genes have been identified for the risk of CHD, it is estimated that 95% of genetic factors remain unidentified in elucidating the pathogenesis of this complex disease (4).

Apolipoprotein A-V (ApoA-V) is a key regulator of TG levels (5,6) and apolipoprotein A5 (*APOA5)* gene variants, including -1131T>C (rs662799) and S19W (rs3135506). These genetic variants have been significantly associated with TG levels (7–9) and the risk of CHD (10). TG levels have been shown to be significantly higher in -1131C controls compared with -1131T controls (7,8). In addition, the -1131C allele frequency of the *APOA5* gene in the early-onset CHD group (43.2%) has been observed to be significantly higher than that in a control group (33.0%) (10). In the S19W variant, the minor allele 19W was found to be rare in the Chinese (0–4.7%) (11) in contrast to that of 15% of the Latin American populations (12). Despite the low minor allele frequency, a positive correlation was found between the 19W allele and CHD in the Chinese population (8,12). In addition, another variant, 553G>T (rs2075291), was shown as a risk factor for CHD in the Han Chinese population (13,14). However, conflicting results of *APOA5* variants were observed in a number of other studies (6,15,16). Studies showed that S19W and -1131T>C were not associated with the risk of CHD in Italian (6) and Brazilian populations (15). The 19W allele was not identified as a risk factor for stroke in Hungarian populations (16). Since previous epidemiological studies indicate that there is an ethnic difference in the *APOA5* gene variants, a meta-analysis of the available data was necessary to investigate the role of the *APOA5* gene in the risk of CHD.

The aim of the current study was to assess whether the *APOA5* gene variants, -1131T>C, S19W and 553G>T, are associated with CHD in the populations studied and to evaluate the contribution of *APOA5* gene variants to CHD in various ethnic populations by meta-analysis.

## Materials and methods

### Sample collection

A total of 819 unrelated individuals were recruited for the case-control study. These included 290 CHD cases, 198 non-CHD controls and 331 healthy controls. CHD cases were patients with >50% coronary artery occlusion of one or more major coronary arteries (17) or a history of prior angioplasty or coronary artery bypass surgery. Non-CHD participants were selected from inpatients who had <50% occlusion in the major coronary artery (18) and did not have any atherosclerotic vascular disease. In addition, 331 apparently healthy individuals from the Ximen Community residents in Ningbo were recruited as healthy controls. CHD cases and non-CHD controls were collected from the Lihuili Hospital (Ningbo, China). CHD cases and non-CHD controls had been examined by standardized coronary angiography according to Seldinger’s method (19) and assessed by at least two independent cardiologists. Subjects were excluded from this study if the individual had congenital heart disease, cardiomyopathy, liver or renal disease or cancer. Blood samples were stored at −80°C until analysis was performed and were treated by the same investigators. The study was approved by the Ethical Committee of Lihuili Hospital in Ningbo (Zhejiang, China) and informed written consent was obtained from all subjects.

### Single nucleotide polymorphism (SNP) genotyping

Human genomic DNA was prepared from peripheral blood samples using the Lab-Aid 820 nucleic acid extraction automatic analyzer (Zeesan Biotech, Xiamen, China) and was quantified using the Quant-iT™ PicoGreen^®^ dsDNA assay kit (Molecular Probes, Inc., Eugene, OR, USA). Amplification was performed on the Geneamp^®^ PCR System 9700 Dual 384-Well Sample Block Module (Applied Biosystems, Foster City, CA, USA) for polymerase chain reaction (PCR). Primers for the single base extension reaction are shown in Table I. The distinct mass of the extended primer indicates different SNP alleles. PCR conditions included an initial denaturation stage at 94°C for 15 sec, followed by 45 cycles at 94°C for 20 sec, 56°C for 30 sec and primer extension at 72°C for 1 min and a final extension for 3 min at 72°C. Primer extension for genotyping was performed on the Sequenom MassARRAY iPLEX^®^ platform (Sequenom, San Diego, CA, USA) according to the manufacturer’s instructions (20). The primer extension reaction included an initial denaturation stage at 94°C for 30 sec, followed by 40 cycles of amplification, including 94°C for 5 sec, 52°C for 5 sec and 80°C for 5 sec, and 5 cycles of amplification, including 52°C for 5 sec and 80°C for 5 sec, and a final extension for 3 min at 72°C. Following purification, the products were subjected to MALDI-TOF mass spectrometry for SNP genotyping using a SpectroCHIP array (Sequenom, San Diego, CA, USA). To verify the repeatability and stability of the experiment, 5% of random samples and 18 control samples, including 9 negative and 9 positive controls, were used for quality control.

### Retrieval of published studies

A search of the studies on *APOA5* gene variants and CHD was conducted in electronic databases, including the Chinese National Knowledge infrastructure, PubMed, Embase, SpringerLink and ScienceDirect, between 2001 and 2012. Specific combinations of keywords were used for the following Medical Subject Heading terms, including ‘coronary heart disease’, ‘coronary artery disease’ or ‘myocardial infarction’ combined with ‘*APOA5*’, ‘apolipoprotein A5’, ‘C56G’, ‘S19W’, ‘-1131T>C’ or ‘553G>T’ and ‘single nucleotide polymorphism’, ‘SNP’ or ‘genetic association’. All studies were considered eligible if they aimed to investigate the correlation between *APOA5* and the risk of CHD. For meta-analysis, studies with one of the following conditions were excluded: i) Studies lacking controls, ii) a lack of detailed main allele or genotype information and iii) duplicate publications.

### Statistical analysis

Departure of Hardy-Weinberg equilibrium (HWE) was analyzed by Arlequin program version 3.5 (21). The allele frequencies and genotype distribution between CHD patients and each of the 2 control groups were compared by CLUMP16 software (Department of Psychological Medicine, Institute of Psychiatry, Denmark Hill, London, UK) with 10,000 Monte Carlo simulations (22). Linkage disequilibrium of *APOA5* gene variants was measured by an online calculator (http://www.oege.orgsoftware/cubex/). Haplotype frequencies were inferred by Arlequin program version 3.5 based on the expectation-maximization algorithm. The odds ratio (OR) with 95% confidence interval (95% CI) was calculated by an online tool (http://faculty.vassar.edu/lowry/odds2x2.html). The statistical power of the study was calculated by the PS power and sample size calculation software version 3.0.43 (23). The correlation between the variants and the severity of CHD was analyzed by the R statistics software (University of Auckland, Auckland, New Zealand). Severity grade of CHD was defined by the number of major coronary arteries with >50% occlusion. Meta-analysis was performed using the Review Manager version 5.1 (The Cochrane Collaboration, The Nordic Cochrane Centre, Copenhagen, Denmark). Heterogeneity of the studies in the meta-analysis was assessed with the Q and I^2^ tests. Publication bias was presented using funnel plots by Review Manager 5.1. The type I error rate was set at 0.05. Two-tailed P<0.05 was considered to indicate a statistically significant difference.

## Results

### Case-control study in Han Chinese populations

SNP S19W was monomorphic in samples of the current study and thus, discarded from further analysis. SNPs -1131T>C and 553G>T were consistent with HWE (P>0.05). As shown in Table II, no significant differences in the 2 SNPs were observed between CHD cases and each of the 2 control groups (P>0.05). Haplotypes of the 2 SNPs were associated with the risk of CHD (data not shown). Under the dominant inheritance model, -1131C was observed to be a CHD risk factor (P=0.030; OR, 1.422; 95% CI, 1.036–1.952; Table III). Further breakdown analysis by gender did not produce significant results between the 2 variants and the risk of CHD (data not shown).

### Correlation between the 2 variants and the severity of CHD

The severity of CHD was defined by the number of coronary arteries with >50% coronary artery occlusion. A logistic regression test was performed between the 2 variants and the severity of CHD in all cases and in a gender-stratified manner. The results indicated that 553G>T correlated with CHD severity in males (Table IV; P=0.032); however, following Bonferroni’s correction, this result was not statistically significant.

### Inclusion of case-control studies for meta-analysis

A total of 23 association studies between *APOA5* gene variants and the risk of CHD were retrieved from the online databases. Among them, 9 studies were excluded from the current meta-analysis as they focused on other *APOA5* variants (24–27) or did not present sufficient information on genotype or allele frequencies or OR values (9,28–31). A total of 14 studies were included in the meta-analysis: -1131T>C, 6,848 cases and 5,452 controls; S19W, 7,644 cases and 10,610 controls; and 553G>T, 4,450 cases and 5,068 controls.

### Meta-analysis of the association studies between -1131T>C and the risk of CHD

**Figure 1:**
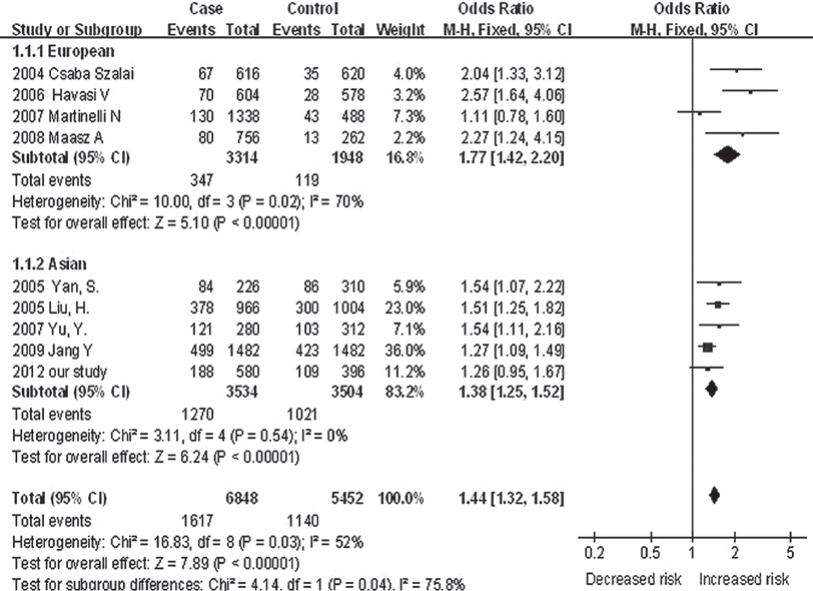
Correlation between rs662799 (-1131T>C) and CHD in the meta-analysis. Events, the number of G alleles; total, total number of A and G alleles; our study, the CHD cases vs. non-CHD controls in our study; CHD, coronary heart disease.

**Figure 2:**
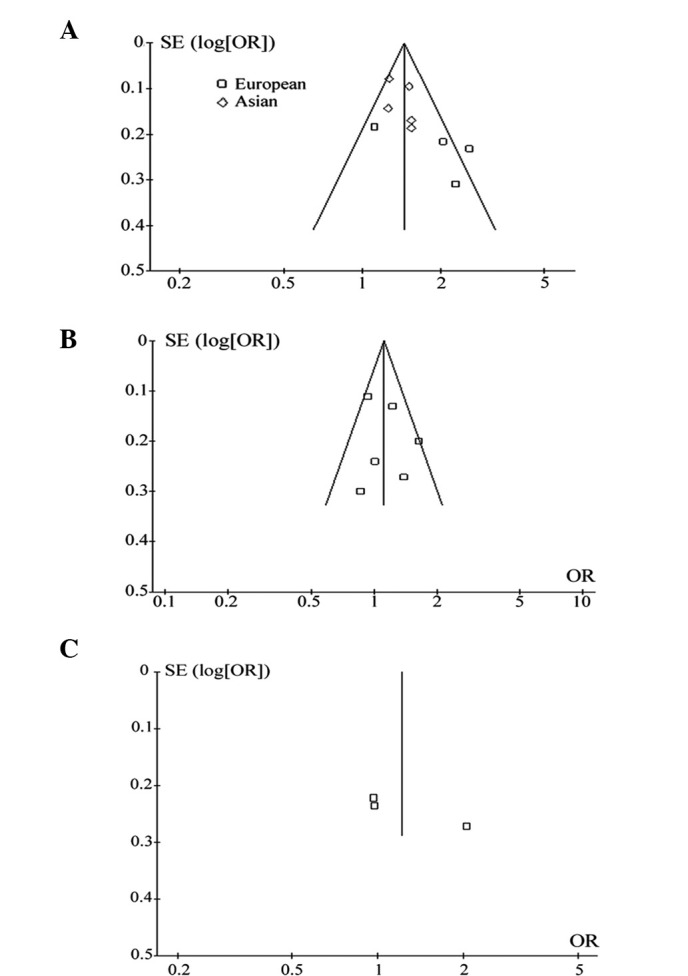
Funnel plot of the 3 SNPs in the *APOA5* gene in the meta-analysis, (A) rs662799 (-1131T>C), (B) rs2075291 (553G>T) and (C) rs3135506 (S19W). *APOA5,* apolipoprotein A5; SNP, single nucleotide polymorphisms.

As shown in Fig. 1, the case-control study (CHD cases vs. non-CHD controls) and 8 other studies (6–8,10,32–35) were included in the meta-analysis. Significant heterogeneity was observed among the 9 studies (I^2^, 52%; χ^2^,16.83; df, 8; P=0.03). Due to the high heterogeneity of these studies, the groups were divided into 2 ethnic subgroups, European and Asian. A significant heterogeneity was observed among Europeans (6,7,33,34) (I^2^, 70%; χ^2^, 10.00; df, 3; P=0.02) in contrast to minimal heterogeneity among Asians (8,10,32,35) (I^2^, 0%; χ^2^, 3.11; df, 4; P=0.54). A significant association between -1131T>C and CHD risk was observed in European individuals (OR, 1.77; 95% CI, 1.42–2.20; P<0.0001) and Asian subgroups (OR, 1.38; 95% CI, 1.25–1.52; P<0.0001). A significant difference was observed between the 2 subgroups (I^2^, 75.8%; χ^2^, 4.14; df, 1; P=0.04). Funnel plot analysis did not reveal publication bias (Fig. 2A).

### Meta-analysis of association studies between 553G>T and the risk of CHD

**Figure 3:**
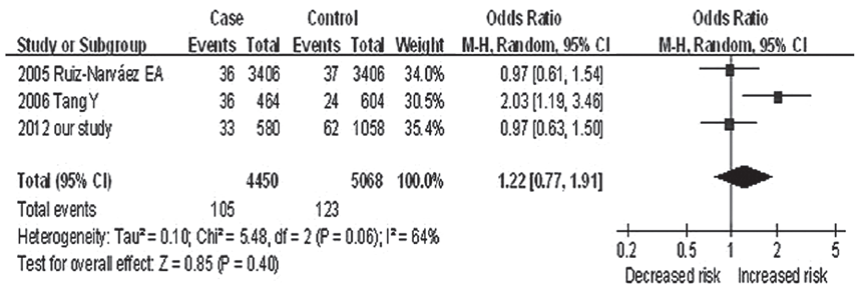
Correlation between rs2075291 (553G>T) and CHD in the meta-analysis. Events, the number of T alleles; total, total number of G and T alleles; our study, the CHD cases vs. diagnosed controls and healthy controls in our study; CHD, coronary heart disease.

Using the fixed effect analysis model, the meta-analysis of 553G>T showed a moderate heterogeneity among the 2 case-control studies (14,36) and the current study (I^2^, 64%; χ^2^, 5.48; df, 2; P=0.06). Due to the moderate heterogeneity, the random effects analysis model was selected for meta-analysis (Fig. 3). The results showed that 553G>T had no significant association with CHD (P=0.40; OR, 1.22; 95% CI, 0.77–1.91). Funnel plot analysis did not reveal publication bias (Fig. 2B).

### Meta-analysis of the association studies between S19W and the risk of CHD

**Figure 4:**
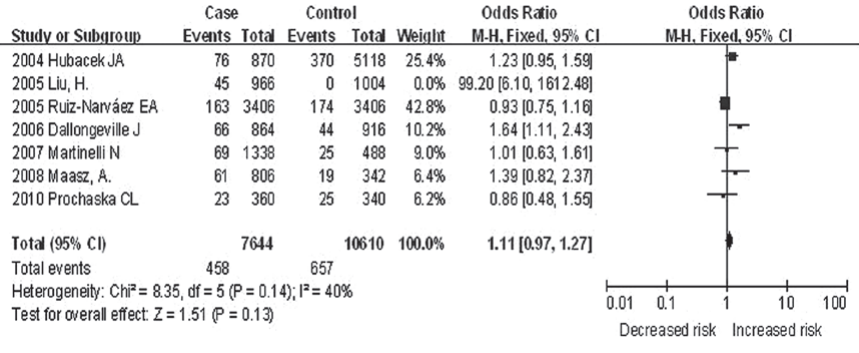
Correlation between rs3135506 (S19W) and CHD in the meta-analysis. Events, the number of C alleles; Total, total number of C and G alleles; CHD, coronary heart disease.

Fig. 4 shows the results of meta-analysis of the associations between S19W and CHD. Since S19W was monomorphic in the samples, only 7 studies were included in the meta-analysis (6,8,15,16,36–38). Under the random effects model, a significantly higher heterogeneity was observed (I^2^, 70%; χ^2^, 19.98; df, 6, P=0.003). An outlier OR-value was observed in 1 study (8) (OR, 99.20; 95% CI, 6.10–1612.48). Following exclusion of the outlier, another meta-analysis was performed and a lower heterogeneity was observed with fixed effect analysis model (Fig. 4; I^2^, 40%; χ^2^, 8.35; df, 5; P=0.14). The results showed that S19W had no significant association with CHD (OR, 1.11; 95% CI, 0.97–1.27; P=0.13). Funnel plot analysis did not reveal publication bias (Fig. 2C).

## Discussion

The *APOA5* gene codes for a 366-amino acid protein, apoA-V, which enhances lipoprotein lipase (LPL) activity (24). Loss of LPL activity interferes with the ability of apoA-V to interact with lipids and lipoproteins, including TGs, very low density lipoproteins and high density lipoproteins (39,40). Elevated plasma TGs are a known risk factor for CHD (41,42) and apoA-V is a major risk factor of CHD as it activates TG hydrolysis in the blood (43). *APOA5* gene variants have been identified as the genetic determinants of TG concentration (9). Since discrepancies exist in previous epidemiological studies on the association of *APOA5* gene variants with CHD, the current study investigated a case-control study in specific populations and meta-analysis of the available case-control data was performed to clarify the role of *APOA5* gene variants in CHD.

SNP -1131T>C is located in the proximal promoter of the *APOA5* gene and is associated with elevated TG levels and hyperinsulinemia (2). A number of studies have found that the -1131T>C gene is significantly associated with CHD in Chinese populations (8,10,32). However, the association between -1131T>C and CHD in the European population remains controversial (6,7,33,34). Three independent studies (7,33,34) observed that patients carrying the -1131CT>C gene had higher TG levels and a significantly increased risk of coronary events. However, Martinelli *et al* demonstrated that 2 *APOA5* variants, including -1131T>C, which are independent predictors of TGs (6), were not associated with CHD (6). The current meta-analysis of 9 studies among 12,300 individuals indicates that -1131CT>C is a risk factor for CHD (pooled OR, 1.44; 95% CI, 1.32–1.58; P<0.00001). An ethnic difference in the prevalence of -1131T>C was observed between the Asian and the European studies (I^2^, 5.8%; P=0.04). The frequency of -1131CT>C in non-CHD controls and healthy controls was 0.380 and 0.376, respectively, which is close to 0.267 in HapMap-HCB and 0.291 in HapMap-JPT (http://www.ncbi.nlm.nih.gov/projects/SNP/snp_ref.cgi?rs=662799). A lower frequency of -1131C was observed in Europeans (0.017 in HapMap-CEU). This is in agreement with the current heterogeneity test results in the meta-analysis.

SNP 553G>T is a rare APOA5 gene variant that has been studied in Han Chinese populations (13,14,29,36). SNP 553G>T has been found to correlate with serum levels of TG and total cholesterol in Han Chinese individuals in Xinjiang, China (29). A significant association between 553G>T and CHD in Han Chinese populations, by 2 separate groups, has been found in Taiwan (P<0.001) and Nanjing (P=0.017) (13,14). In the present study, the previous positive association between 553G>T and CHD was not observed. A meta-analysis of 3 studies among 9,518 individuals indicates that the 553G>T gene is not associated with CHD risk (P=0.40; OR, 1.22; 95% CI, 0.77–1.91). However, a correlation between 553G>T and the severity of CHD was observed in males by a logistic regression analysis (P=0.032). Further investigation of the contribution of 553G>T to the progression of CHD is required.

The minor allele frequency of S19W in Chinese populations was significantly different from that in Caucasians. The 19W allele is rare in HapMap-CHB (0%), thus providing an explanation as to why S19W was monomorphic in the samples, including 290 CHD cases, 198 non-CHD controls and 331 healthy controls. Liu *et al* observed a 4.7% prevalence of 19W in the CHD cases, while in Chinese populations, 19W was not observed (0%) (8). However, another case-control study in Chinese populations did not observe 19W in cases and controls and thus, hypothesized a negative association between SNP 19W and CHD (11,44). The allele frequency of 19W was 0.1% in Chinese Singaporean populations (12). However, 19W was more common in Europeans (HapMap-CEU: 5.8%). The allele frequencies of 19W in Malay and the Asian-Indian populations were 1.7 and 3.1%, respectively, while in Latin-American populations, allele frequency was 15% (12). The current meta-analysis of 6 studies (6,15,16,36–38) among 18,254 individuals found no significant association between CHD and S19W (P=0.13; OR, 1.11; 95% CI, 0.97–1.27).

In summary, the current case-control study shows that the -1131CT>C gene is a CHD risk factor in the populations studied and this association was further supported by meta-analysis. The case-control study has <80% statistical power (the strongest power observed for -1131T>C was 58.8%). An improved case-control investigation with larger sample sizes and a balanced gender structure is required in the future.

## Figures and Tables

**Table I:** Primer sequences for single base extension reaction.

SNP	Name	Primer	Sequence (5′-3′)
rs662799	-1131T>C	1st-P	ACGTTGGATGGCCCTGCGAGTGGAGTTCA
		2nd-P	ACGTTGGATGACTCTGAGCCCCAGGAACT
		UEP_SEQ	GGGTGAACTGGAGCGAAAGT
rs3135506	S19W	1st-P	ACGTTGGATGTGGTCTGGCTGAAGTAGTCC
		2nd-P	ACGTTGGATGTGATTACCTAGTCCCTCTCC
		UEP_SEQ	TAGGCCCTCTCCACAGCGTTTT
rs2075291	553G>T	1st-P	ACGTTGGATGTTGGGCTTTGCTGCAGGGAC
		2nd-P	ACGTTGGATGATGGGTGGAAGAGCTCTTTG
		UEP_SEQ	GCTCTTTGAAGCGGC

SNP, single nucleotide polymorphism.

**Table II:** Frequencies of the genotype and allele for SNPs.

A, 553G>T											

Total	Genotype (n)			χ^2^	P-value, df=2	HWE	Allele (n)		χ^2^	P-value, df=1	OR (95% CI)
	
	GG	GT	TT				G	T			
CHD cases, n=290	258	31	1			1.000	547	33			
Non-CHD controls, n=198	169	29	0	2.357	0.261	0.605	367	29	1.056	0.352	0.764 (0.456–1.279)
Healthy controls, n=331	299	31	1	0.312	0.799	0.567	629	33	0.305	0.614	1.150 (0.700–1.888)

B, -1131T>C											

Total	Genotype (n)			χ^2^	P-value, df=2	HWE	Allele (n)		χ^2^	P-value, df=1	OR (95% CI)
	
	AA	AG	GG				A	G			

CHD cases, n=290	134	124	32			0.685	392	188			
Non-CHD controls, n=198	106	75	17	2.675	0.257	0.476	287	109	2.656	0.107	1.263 (0.954–1.672)
Healthy controls, n=331	182	117	32	4.808	0.090	0.051	481	181	3.809	0.054	1.275 (0.999–1.626)

SNP, single nucleotide polymorphism; HWE, Hardy-Weinberg equilibrium; OR, odds ratio; CI, confidence interval; CHD, coronary heart disease.

**Table III:** Significant differences in genotype distributions under the dominant model.

	CHD cases vs. non-CHD controls		CHD cases vs. healthy controls	
		
Dominant model	OR (95% CI)	P-value, df=1	OR (95% CI)	P-value, df=1
Total				
rs2075291 (GT + TT vs. GG)	0.723 (0.422–1.239)	0.266	1.159 (0.691–1.945)	0.599
rs662799 (AG + GG vs. AA)	1.341 (0.934–1.927)	0.118	1.422 (1.036–1.952)	0.030
Male				
rs2075291 (GT + TT vs. GG)	0.638 (0.325–1.249)	0.211	1.720 (0.677–4.370)	0.295
rs662799 (AG + GG vs. AA)	1.343 (0.834–2.163)	0.229	1.499 (0.903–2.488)	0.126
Female				
rs2075291 (GT + TT vs. GG)	0.787 (0.305–2.031)	0.643	0.936 (0.406–2.159)	1.000
rs662799 (AG + GG vs. AA)	1.515 (0.834–2.753)	0.178	1.664 (0.998–2.774)	0.054

CHD, coronary heart disease; OR, odds ratio; CI, confidence interval.

**Table IV:** Logistic regression analysis of association of SNPs and the serious extent of CHD disease.

Parameters	Non-CHD controls	One artery	Two arteries	≥Three arteries	rs2075291	rs662799
Total	**198**	**106**	**65**	**119**	*0.091*	*0.283*
Male	**101**	**77**	**49**	**84**	*0.032*	*0.568*
Female	**97**	**29**	**16**	**35**	*0.898*	*0.219*

Numbers in bold represent cases of patients under the corresponding conditions, numbers in italic represent P-values which indicate the association of the SNPs with the serious extent of disease. SNP, single nucleotide polymorphism; CHD, coronary heart disease.

## Abbreviations

SNP: single nucleotide polymorphism
CHD: coronary heart disease
HWE: Hardy-Weinberg equilibrium
GWAS: genome-wide association studies
*APOA5*: apolipoprotein A5
TG: triglyceride
OR: odds ratio
CI: confidence interval
